# Evaluation of Cholinesterase Activities During *in Vivo* Intoxication Using an Electrochemical Sensor Strip – Correlation With Intoxication Symptoms

**DOI:** 10.3390/s90503627

**Published:** 2009-05-14

**Authors:** Miroslav Pohanka, Ladislav Novotný, Jan Misík, Kamil Kuca, Jana Zdarova-Karasova, Martina Hrabinova

**Affiliations:** 1 Centre of Advanced Studies, Faculty of Military Health Sciences, University of Defense, Hradec Kralove, Czech Republic; E-Mail: novotnyl@pmfhk.cz (L.N.); 2 Department of Toxicology, Faculty of Military Health Sciences, University of Defense, Hradec Kralove, Czech Republic; E-Mail: honzamisik@seznam.cz (J.M.); kucakam@pmfhk.cz (K.K.)

**Keywords:** cholinesterase, intoxication, acetylcholinesterase, butyrylcholinesterase, paraoxon, pesticide, activity, blood

## Abstract

Cholinesterase activity in blood of laboratory rats was monitored. Rats were intoxicated with paraoxon at dosis of 0 – 65 – 125 – 170 – 250 – 500 nmol. The 250 nmol dose was found to be the LD_50_. An electrochemical sensor was found useful to provide information about cholinesterase activity. The decrease of cholinesterase activity was correlated to intoxication symptoms and mortality level. It was found that the symptoms of intoxication are not observed while at least 50% of cholinesterase activity in blood remains. The minimal cholinesterase activity essential to survival is around 10%, when compared with the initial state. No changes in levels of low moleculary weight antioxidants were observed.

## 1. Introduction

Many pesticides are known to be neurotoxic. One group of them, the organophosphates, are strong irreversible inhibitors of two important enzymes in the organism: acetylcholinesterase (AChE; EC 3.1.1.7) and butyrylcholinesterase (BChE; EC 3.1.1.8). The most important role of AChE is terminating neurotransmission by hydrolysis of acetylcholine [1]. Inhibition of AChE is based on bonding to serine in the active site [2]. The *in vivo* inhibition results in accumulation of acetylcholine inside neurosynapses with consequent overstimulation of acetylcholine receptors [3].

Many symptoms can occur *in vivo* shortly after intoxication. Typical intoxication symptoms should be considered a consequence of overstimulation of muscarinic and/or nicotinic acetylcholine receptors [4]. Typical symptoms are bronchospasms, bradycardia, miosis, lacrymation, diarrhea and salivation. Moreover, typical symptoms of CNS nicotinic and muscarinic receptor overstimulation can occur: confusion, coma, agitation and/or respiratory failure [4].

Typically examination of cholinesterase activity in blood is based on Ellman's reaction [5,6]. It is based on splitting of an artificial substrate acetylthiocholine into acetic acid and thiocholine and consequent reaction with 5,5′-dithiobis-2-nitrobenzoic acid. The accumulation of 5-thio-2-nitrobenzoic acid is measured as absorbance at 412 nm. The disadvantage of Ellman's method is the strong interference caused by many electrophilic compounds such as reactivator drugs with oxime groups [7]. Voltammetric techniques have been found useful for routine assays of biological matrices [8-11]. The performance of electrochemical devices has been found convenient to assay anticholinergic compounds such as nerve agents, pesticides and some drugs [12-14]. Cholinesterase is bound tightly to the electrode surface, so the resulting device is considered a biosensor [15-16]. Recently, the electrochemical assay of blood cholinesterases was proposed as a plausible alternative to the optical one [17].

Though the mechanism of intoxication has been extensively studied for the last decades, to the best of our knowledge the estimation of the exact levels of cholinesterase activity necessary for survival has not been established. The study is focused on evaluation of blood cholinesterase activity during serious intoxication by the organophosphate paraoxon. The data are correlated with mortality and symptomatic manifestations of intoxication. An electrochemical sensor was used in these experiments for biochemical examination of cholinesterases as a practical alternative capable of providing unique data.

## 2. Results and Discussion

Animals were intoxicated with a wide range of paraoxon concentrations. Symptomatic manifestation was taken not only as a measure of successful intoxication, but also as a parameter subsequently correlated to the cholinesterase activity. Any resulting fast mortality was studied as another important parameter, but on the other hand, since animals were sacrificed after half an hour any pertinent mortality could not be evaluated after this interval and final mortality over a long term period could be quite different. The experiment was aimed at the acute phase of intoxication. This period is crucial since treatment by reactivators such as obidoxime and partially HI-6 are effective for paraoxon [18,19]. After that, no treatment is possible due to dealkylation of organophosphate inside reactive site i.e. aging [20,21]. Activity of blood cholinesterases is considered as important marker of intoxication by organophosphates. The primary effect of blood AChE, as part of the cell signaling system, is different to the one from neurosynapses. In particular, AChE is associated on macrophages with nicotinic cholinergic receptors inhibiting TNF synthesis and modulating inflammation [35,36]. On the other side, a common role of BChE in the body is not still fully recognized. BChE is considered as part of the body's defense mechanisms, able to hydrolyzing toxic compounds such as cocaine [37].

### 2.1. Mortality and symptoms

Mortality is one final effect of toxins. The experiment was planned in way to cover a full spectrum of mortality. Rats used in experiments were sacrificed within thirty minutes after intoxication. This short period of paraoxon incidence should ensure that any observations are due to the acute toxicity and cholinergic crisis phases, rather than other mechanisms such as apoptosis arising later [22].

No mortality was observed up to a dose of 170 nmol/kg of body weight (b.wt.). Above 170 nmol, the mortality rose abruptly. A dose of 250 nmol/kg b.wt. of paraoxon was found to be the LD_50_ for the given time interval. Doubling the LD_50_ dose up to 500 nmol/kg b.wt. of paraoxon led to overall mortality within ten minutes. Symptomatic manifestations of intoxication were observed starting at the 170 nmol/kg b.wt. dose. Some animals exposed to this dose manifested tonic-clonic seizures, but the symptomatic manifestations caused by the 170 nmol/kg b.wt. dose was slight in comparison with a 250 nmol/kg b.wt. dose. The latter caused strong abrupt tonic-clonic seizures within 5 minutes and overall deterioration of shape. The deaths occurred after quite a long period: 20 minutes. The highest tested dose was 500 nmol/kg b.wt. of paraoxon per animal. Tonic-clonic seizures were of similar level as observed at a dose of 250 nmol/kg b.wt, however animals in this cohort manifested tonic-clonic seizures within 5 minutes and died within 10 minutes after paraoxon intoxication. No other specific symptoms were clearly visible. Every exitus was confirmed by proven cardiac arrest and persisting mydriasis. The symptomatic manifestation observed during experiment correlates with the expected from human cases [23].

### 2.1. Cholinesterase activity

Cholinesterase activity was assayed in the way described previously [17]. Faraday's laws of electrolysis were used to calculate cholinesterase activity. Current measured in the reaction mixture grew linearly for approximately 5 minutes. The total electric charge flowed through the system was estimated from the area under the curve. The total flowed charge was simply recalculated to give enzyme activity. The resulting data are summarized in Table 2.

The level of cholinesterases in blood is an important marker of intoxication and strongly correlates with AChE level in CNS as well as PNS [24, 25]. Here, a deterioration of overall shape was observed when at least 50% of cholinesterase activity is inhibited. This corresponded to a dose of less than 170 nmol/kg b.wt. Cholinergic crises happened when more than 80% of cholinesterases were inhibited. This responded at 170 nmol/kg b.wt. of paraoxon. Deaths were observed when inhibition surpassed 90%. This corresponded to doses of 250 and 500 nmol/kg b.wt. Death was caused by a dose of 500 nmol/kg b.wt. per animal. The corresponding inhibition level was nearly 95%. The differences were statistically relevance (ANOVA with Scheffe test, Origin 8 software, OriginLab). Only cholinesterasese activity levels caused by doses renging from 65 – 125 nmol/kg b.wt. and doses 250 – 500 nmol/kg b.wt. were indistinguishable from each other at a probability level P = 0.05. All other groups were found different with each other at a probability level P = 0.05.

The examination of total levels of low molecular weight antioxidants was used as a marker of resulting oxidative stress. The anodic wave showed no significant change for any assayed plasma sample. The achieved current was equal to 2,940 ± 154 nA at a voltage of 665 ± 48 mV. The data suggest no oxidative stress in the laboratory animals during the experiments. It seems that the paraoxon toxicology pathway was based only on short term neurotoxicity.

This study suggests a quite extensive tolerance of organisms to small decreases in cholinesterase levels. On the other hand, the findings of acute poisoning by organophosphate are not useful for assessing shock states and consequences over a long term [26]. Slight inhibition of cholinesterases could be even positive in oxidative stress conditions, for example, by reducing e.g. interleukin-1 beta in Alzheimer's disease [27] and bronchoconstriction during anaphylactic shock [28]. Many drugs such as tacrine and its derivates are also based on inhibition of AChE [14, 29]. Here, we propose that body possesses the capability to resist overdosing by anticholinergic compounds at quite high levels. It is an important fact, when we consider the limited efficacy of currently used antidotes [30] and their limited penetration inside the brain [31].

## 3. Experimental Section

### 3.1. Animals

Male Wistar rats were purchased from Velaz Ltd. (Prague, Czech Republic). Animals were of the same age, weighing 250 – 300 g. The animals were maintained under SATP conditions and 50±10% humidity. Illumination lasted from 7 a.m. to 7 p.m. Animals were allowed to ingest chow and water without any restriction. Animals were housed in the Animal House of the Faculty of Military Health Sciences in Hradec Kralove. Maintenance of animals and all experiments were supervised by the Ethical Committee of the Faculty of Military Health Sciences and a veterinary surgeon.

### 3.2. Animal intoxication

The laboratory rats were intoxicated with paraxon via intramuscular injection into the caudal thigh muscles. The paraoxon has been diluted with saline to a volume of 200 μL. This volume was not exceeded in order to prevent backward discharge. The weight of individual animals was taken into account when the paraoxon solutions were prepared. Each animal was been unmistakable marked and attentively observed. Every clinical symptom was registered.

### 3.3. Blood processing and amperometric evaluation of enzyme activity

Amperometric principles were used for evaluation of cholinesterase activities. Freshly collected blood was mixed with 0.02 M Tris buffer pH 7.6 in a 1:20 ratio. The mixture was left to incubate for 5 minutes in laboratory temperature. No clouds were observed after cell disruption by Tris buffer. The mixtures were not centrifuged in order to retain activity without any losses. Blood lysate (0.5 mL) was mixed with 1 mM acetylthiocholine chloride (ATChCl, 1.5 mL) in phosphate buffered saline (PBS). The mixture was placed into a reaction cell and mixed with a magnetic stirrer coated with Teflon. An electrochemical sensor strip with platinum working and platinum auxiliary electrodes and silver covered by silver chloride as reference electrode (BVT Brno, Czech Republic) was used throughout the experiments for measuring enzyme activity. The sensor was connected to an EmStat device (Houten, The Netherlands) and the applied voltage between working and reference electrode was adjusted up to +500 mV. Since the measuring principle was chronoamperometry, voltage was constant for the all experiments. The current was measured as described in the literature [17]. Percent of inhibition (I) is the percent decrease of cholinesterase activity in comparison with intact blood. It was calculated according to reference [12].

### 3.4. Cyclic voltammetry

Cyclic voltammetry was performed in order to estimate the total level of low molecular weight antioxidants (LMWA). The cited studies were slightly adapted to perform the assay [24, 32-34]. The electrochemical sensor (the same type as above) was fixed horizontally and the electrodes were overlaid with 20 μL of undiluted plasma. Cyclic voltammetry was measured in a range -0.5 to 1.1 V with a scan rate of 50 mV/s.

## 4. Conclusions

Cholinesterase activity is an important biochemical parameter. Though the activity was clearly correlated to organophosphate intoxication, the symptomatic manifestations as well as mortality resulted from at higher dose of paraoxon. It seems that the minimal level of cholinesterase activity to ensure survival is 90%. The precise evaluation of cholinesterase activity was possible with a sensor method based on electrochemical measurement of accumulated thiocholine. The achieved data would be found as an useful algorithm for humans intoxicated by organophosphates; especially, the seriousness of intoxication would be estimated by biochemical examination of blood cholinesterases.

## Figures and Tables

**Table 1.** Mortality progression as the increased dose of paraoxon administered into rats.

**Dose (nmol/kg b.wt.)**	**Mortality (%)**	**Symptoms**
0	0	No
65	0	No
125	0	No
170	0	Slight tonic-clonic seizures after 10 minutes, only in quarter of the cohort
250	50	Tonic-clonic seizures at the whole cohort after 10 minutes. Mortality seen 20 minutes after administration of paraoxon in a quarter of the cohort.
500	100	Tonic-clonic seizures within 5 minutes after administration. Mortality of the whole cohort within 10 minutes.

**Table 2.** Changes in cholinesterase activity after exposition to paraoxon. Letter I indicates percent of cholinesterases inhibition.

**Dose (nmol/kg b.wt.)**	**activity (μkat/l)**	**I(%)**
0	656 ± 105	/
65	343 ± 24	47.7±4.1
125	309 ± 70	52.9±11.6
170	90 ± 11	86.3±2.1
250	62 ± 49	90.5±5.4
500	34 ± 25	94.8±2.9
